# Pressurized Intra-Peritoneal Aerosol Chemotherapy (PIPAC) for Peritoneal Malignancies with Palliative and Bidirectional Intent

**DOI:** 10.3390/cancers17121938

**Published:** 2025-06-11

**Authors:** Daniele Marrelli, Ludovico Carbone, Daniele Fusario, Roberto Petrioli, Gianmario Edoardo Poto, Giulia Grassi, Riccardo Piagnerelli, Stefania Angela Piccioni, Carmelo Ricci, Maria Teresa Bianco, Maria Antonietta Mazzei, Stefano Lazzi, Franco Roviello

**Affiliations:** 1 Unit of General Surgery, Department of Oncology, AOUS, 53100 Siena, Italy; ludovicocarbone1@gmail.com (L.C.); gianmarioepoto@gmail.com (G.E.P.);; 2 Unit of Medical Oncology, Department of Oncology, AOUS, 53100 Siena, Italy; r.petrioli@ao-siena.toscana.it; 3 Interventional Radiology Unit, Department of Emergency, AOUS, 53100 Siena, Italy; 4 Hygiene Prevention and Protection Area, Pharmacy Unit, AOUS, 53100 Siena, Italy; m.bianco@ao-siena.toscana.it; 5 Unit of Diagnostic Imaging, Department of Medicine Surgery and Neurosciences, University of Siena, 53100 Siena, Italy; 6 Pathological Anatomy Unit, Department of Medical Biotechnology, University of Siena, 51300 Siena, Italy

**Keywords:** pressurized intraperitoneal aerosol chemotherapy, PIPAC, peritoneal surface malignancies, peritoneal metastases, locoregional chemotherapy, neoadjuvant treatment, conversion, bidirectional

## Abstract

**Background**: PIPAC is an innovative treatment that delivers low-dose aerosolized chemotherapy into the abdominal cavity of patients with peritoneal surface malignancies (PSMs). However, its role in the multimodal management of PSMs is unclear. **Methods**: We retrospectively analyzed data from 64 patients who underwent PIPAC for PSMs of a primary or secondary origin between June 2020 and December 2024 (median age of 64 years). Primary tumor sites included gastric (42.2%), colorectal (23.4%), ovarian cancer (21.9%), and others (12.5%). The median PCI was 15 (IQR 9–25), with ascites present in 60.9% of cases and a positive cytology in 48.4%. **Results**: A total of 82 PIPAC sessions were performed in 64 patients. The mean operation time was 96 min. Severe adverse events, defined as the Common Terminology Criteria for Adverse Events (CTCAE) of a grade ≥ 2, occurred in four patients (6.2%). The median hospital stay was 3 days, and systemic chemotherapy was resumed within 14 days after the procedure in 27 patients. Among the entire cohort, 37.5% received bidirectional therapy and 62.5% received palliative treatment, with a lower peritoneal cancer index (PCI) in the bidirectional group (9.5 vs. 23). The median overall survival (OS) was 32 months from diagnosis. Sixteen patients (25%) underwent two or more PIPAC sessions and showed an advantage in survival compared to patients who underwent only one procedure (3-year OS: 63.2% vs. 38.4%, *p* 0.030). Conversion surgery was achieved in 34.4%. Patients treated with a bidirectional intent demonstrated a longer OS (3-year: 66.0% vs. 33.9%, *p* 0.011). Colorectal and ovarian tumors exhibited better long-term outcomes compared to gastric cancer. **Conclusions**: PIPAC is a promising treatment for PSMs, with a low morbidity rate. Its favorable safety and short interval to systemic therapy resumption support its use as part of a bidirectional strategy.

## Simple Summary

Patients with tumors that have spread to the peritoneum often face limited treatment options and poor outcomes. Pressurized Intraperitoneal Aerosol Chemotherapy (PIPAC) is a novel, minimally invasive technique that delivers chemotherapy directly into the abdominal cavity with fewer side effects. This study included 64 patients with tumors of various origins who underwent PIPAC, either as a palliative measure or as part of a bidirectional treatment strategy. We found that PIPAC was generally safe, with a severe complication rate of 6.2%. Patients who received more than one PIPAC session or were treated with a bidirectional approach had improved survival outcomes. Additionally, 22 patients underwent cytoreductive surgery with a potentially curative intent. These findings support the further investigation of PIPAC as a promising option in the multimodal treatment of advanced abdominal cancers.

## 1. Introduction

The treatment of peritoneal surface malignancies (PSMs) remains challenging, mainly due to their poor prognosis [1,2]. Palliative systemic chemotherapy has shown limited effectiveness. The peritoneal–plasma barrier reduces the concentration of systemically delivered drugs in the peritoneal cavity, which often results in minimal therapeutic benefit and poor survival outcomes.

To address this limitation, locoregional therapies have been developed [3,4]. Pressurized Intraperitoneal Aerosol Chemotherapy (PIPAC) is a minimally invasive technique that administers low-dose chemotherapy as a pressurized aerosol into the abdominal cavity. PIPAC offers multiple advantages: (a) an improved drug distribution throughout the virtual recesses of the abdomen due to aerosolization; (b) deeper tissue penetration and a prolonged local drug exposure; (c) a reduced systemic toxicity thanks to its limited absorption; and (d) the possibility of repeating the procedure, with, additionally, an adequate assessment of the response to the treatment [5,6]. Moreover, as part of a (re)staging laparoscopy, PIPAC does not include visceral resections and is generally associated with a lower morbidity [7].

PIPAC was introduced in 2011 as a palliative treatment modality for managing malignant ascites in patients with PSMs of a gastric, ovarian, and colorectal origin [8,9,10]. More recently, its application has been extended to include patients with unresectable PSMs of a hepato-biliary or pancreatic origin, as well as those with mesothelioma [11,12]. There is an increasing interest in combining PIPAC with systemic chemotherapy within a bidirectional therapeutic approach, whereby repeated intraperitoneal administrations are integrated with intravenous chemotherapy. This strategy aims to facilitate the downstaging of the disease in patients initially deemed unresectable, potentially enabling a delayed cytoreductive surgery (CRS) combined with hyperthermic intraperitoneal chemotherapy (HIPEC) [13,14]. Nevertheless, a careful patient selection at the initiation of the PIPAC program remains imperative to maximize therapeutic efficacy and minimize treatment discontinuation. Definitive survival outcomes are lacking, and numerous international guidelines have yet to incorporate PIPAC as a standard treatment option.

In the present study, a consecutive cohort of patients undergoing PIPAC with a palliative or bidirectional approach were analyzed.

## 2. Materials and Methods

Data was extracted from a prospectively maintained PIPAC database from June 2020 to December 2024.

### 2.1. Patients and Measures

Patients aged > 18 years and diagnosed with primary or secondary PSM were discussed in a multidisciplinary team to determine optimal patient selection and the appropriate therapeutic management. Preoperative Eastern Cooperative Oncology Group (ECOG) performance status and Prior Surgical Score (PSS) were calculated as suggested by Sugarbaker [15]. Demographic information and preoperative characteristics, including blood tests, cardiovascular and respiratory function assessments, were assessed to rule out any contra-indications for surgery, anesthesia, or chemotherapy administration.

Criteria for eligibility were as follows: (a) patients with PSM who had not received treatment or who exhibited a sort of response to systemic chemotherapy (partial, stable, or mild progression)—cases with rapid progression during chemotherapy were considered as not eligible for PIPAC; (b) patients not eligible for CRS and HIPEC due to the extensive dissemination of disease; and (c) ECOG performance status equal or less than 2. Exclusion criteria were as follows: (a) presence of extra-abdominal or hematogenous metastases; (b) bowel occlusion, inability to oral feed, or the need for parenteral nutrition; and (c) impaired liver or renal function, portal vein thrombosis, severe hypersensitivity reactions to platinum or doxorubicin, and medical contra-indications to insufflation into the peritoneal cavity. PIPAC was delivered in most patients as a combined treatment with systemic chemotherapy. All included patients were submitted to one or repeated sessions of PIPAC. During the procedure, the peritoneal cancer index (PCI) was routinely scored for preoperative assessment of peritoneal disease extent [16].

Two groups of patients were identified in the present study: (1) bidirectional group—patients with primary diagnosis of unresectable peritoneal carcinomatosis of various origins, generally synchronous, in which a therapeutic course of systemic chemotherapy, combined with one or more cycles of PIPAC, was planned; the main aim was to downstage peritoneal carcinomatosis and lead patients to CRS and HIPEC; (2) palliative group—all other cases; in this group, patients submitted to more lines of chemotherapy, recurrent peritoneal carcinomatosis, or massive ascites, were included.

Patients with major or complete intraperitoneal tumor regression assessed by radiological response according to Response Evaluation Criteria in Solid Tumors (RECIST) criteria, or staging laparoscopy performed during PIPAC treatment, were programmed to a conversion surgery [17].

### 2.2. PIPAC Procedure

All PIPAC procedures were performed laparoscopically in accordance with the established safety protocols, as recommended [18].

**Figure 1:**
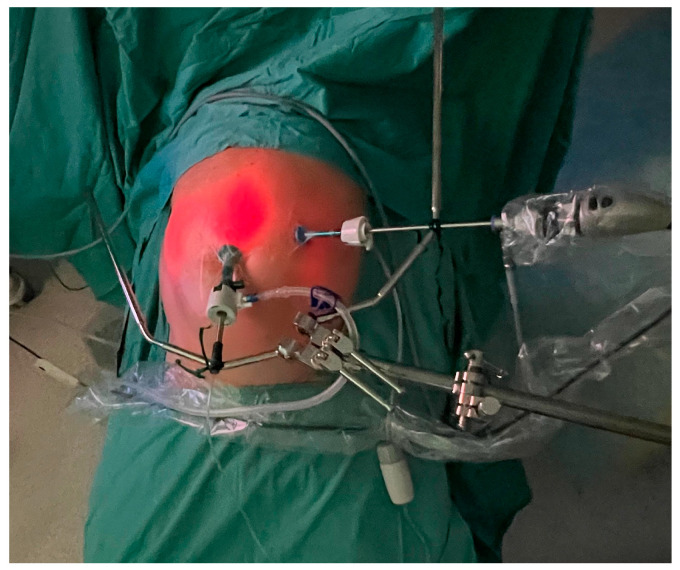
The trocar placement: a 5 mm balloon trocar with a laparoscope placed on the left side and a 10 mm balloon trocar with a CE-certified nebulizer connected to a high-pressure injector in the lower abdomen.

Under general anesthesia, a pneumoperitoneum was established at a pressure of 12 mmHg by means of an open insertion of a 10 mm balloon trocar (Applied Medical, Paris, France). One or two additional balloon trocars were subsequently placed under direct visualization (Figure 1).

**Figure 2:**
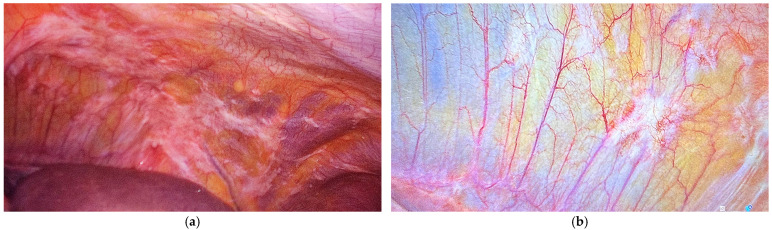
The left upper region in a patient with gastric cancer and synchronous peritoneal metastases (PCI 12): (**a**) the staging laparoscopy before PIPAC and (**b**) the restaging after PIPAC (peritoneal biopsies were negative for neoplasms during the histopathological examination).

A staging laparoscopy was then performed, including PCI assessment and video and photographic records. Representative parietal peritoneal nodules were biopsied, and ascites, when present, were quantified, aspirated, and submitted for cytological analysis. In absence of ascites, a cytological examination of peritoneal washing was always performed (Figure 2).

Prior to the administration of chemotherapy, adherence to the safety protocol was confirmed using a tailored checklist addressing both team- and procedure-specific safety measures [19,20]. Chemotherapeutic agents were doxorubicin at a dose of 1.5 mg/m^2^ body surface area in 50 mL of 0.9% NaCl and cisplatin at a dose of 7.5 mg/m^2^ body surface area in 150 mL of 0.9% NaCl [21]. Drugs were aerosolized using a CE-certified nebulizer (CapnoPen, Capnomed, Villingendorf, Germany) connected to a standard intravenous high-pressure injector. This delivered the solution at a rate of 0.7 mL/s with a maximum pressure of 220 psi. Following chemotherapy administration, a steady-state pneumoperitoneum was maintained for 30 min. Intra-abdominal gas was then evacuated via a Closed Aerosol Waste System equipped with two microparticle filters to capture residual chemotherapeutic particles.

Postoperative pain management was guided by the Numeric Pain Rating Scale and involved the administration of either paracetamol or non-steroidal anti-inflammatory drugs as appropriate.

Two patients, for whom access to the abdomen was not possible due to severe adhesions resulting from previous operations, were excluded from the analysis.

### 2.3. Endpoints

Safety and efficacy of PIPAC were evaluated. Safety was attempted based on the incidence of severe adverse events, as classified by the Common Terminology Criteria for Adverse Events (CTCAE), version 4.0 [22]. Efficacy was evaluated through the assessment of disease response rate, the rate of conversion to surgery, and overall survival (OS). OS was defined as the time from the date of diagnosis, or the time from the first PIPAC, to death or last follow-up time, irrespective of the cause of death. None of included patients were lost at follow-up.

### 2.4. Statistics

Categorical variables were reported as numbers and percentages. Continuous variables were reported as mean and standard deviation (SD) or median and interquartile range (IQR), depending on the normal distribution of the data. Where applicable, group comparisons were conducted using Fisher’s exact test for categorical variables and the Mann–Whitney U test for continuous variables. A two-tailed *p*-value of <0.05 was considered statistically significant. Survival analyses were plotted using the Kaplan–Meier survival estimator. The statistical software SPSS, version 27, (Stata Corp, College Station, TX, USA) was used for statistical analyses.

## 3. Results

Our cohort consisted of 64 patients (22 males and 42 females) who underwent 82 total procedures. The median age was 64 (IQR 56–73) and the mean BMI was 24.3 (SD 4.4). The ECOG performance status was zero or one in 82.8%.

### 3.1. Clinical

The procedure was carried out after a mean interval of 10 months (IQR 3–15) following the diagnosis of the PSM. The primary tumor site was gastric in 27 patients (42.2%), colorectal in 15 (23.4%), ovarian in 14 (21.9%), pancreatic in 4 (6.2%), mesothelioma in 3 (4.7%), and the breast in 1 case (1.6). The PSS was zero, one, two, and three in 46 (71.9%), 5 (7.8%), 6 (9.4%), and 7 (10.9%) patients, respectively.

### 3.2. Surgical

The median PCI at surgery was 15 (IQR 9–25). Ascites was drained in 39 (60.9%) cases and a positive cytology was found in 31 (48.4%). The Pathological Peritoneal Regression Grading Score after the first PIPAC session was two (isolated or small clusters) in three (18.8%) cases, while scores of three (predominant over fibrosis) or four (visible tumor cells at lowest magnification) were observed in thirteen cases (81.2%) [23].

Patients were divided into two groups: 24 (37.5%) having a PIPAC session with a bidirectional intent and 40 (62.5%) undergoing palliative treatment. Cases treated with a bidirectional intent had a lower PCI at the laparoscopy (9.5 vs. 23; *p* < 0.001).

The mean duration of surgery was 95.7 (SD 20.8) minutes. Sixteen patients underwent two or three sessions.

Postoperative complications, classified according to the CTCAE, included five cases of bone marrow hypocellularity (Grade I), one case of electrolyte imbalance (Grade II), one mild–severe anemia case requiring a transfusion (Grade III) and two cases of infectious complications (Grade III), specifically a case of cholangitis and a case of sepsis related to the central venous access port.

The median hospitalization time was 3 (IQR 2–4) days. The systemic chemotherapy was administered within 14 days following the procedure in 27 patients.

Two patients died within 30 days following hospital discharge, both in the palliative group (Table 1).

### 3.3. Survival

**Figure 3:**
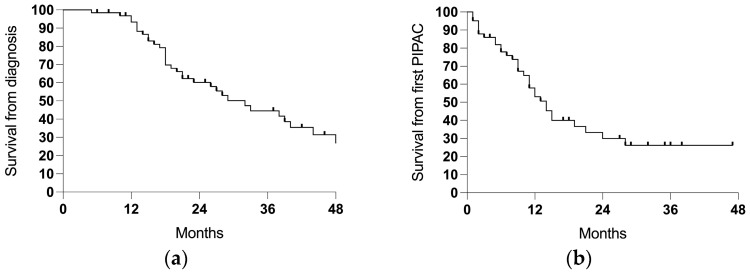
The survival in the entire cohort (64 cases): (**a**) the survival from the diagnosis of the PSM and (**b**) the survival from the first PIPAC session.

The median follow-up time was 21 months (range 5–69) from the diagnosis of the PSM, with a median survival of 32 months and a 3-year survival rate of 44.6% (Table 2; Figure 3a). Counting from the first PIPAC session, the median follow-up time was 9 (1–28) months and the median survival was 14 months (Figure 3b).

**Figure 4:**
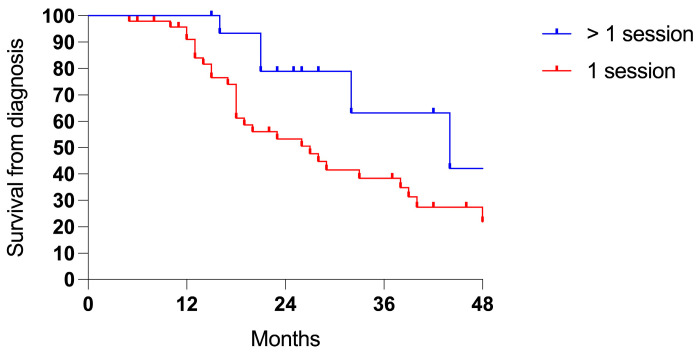
Survival comparing one (48 cases) vs. two or more PIPAC sessions (16 cases).

Patients receiving more sessions of PIPAC showed an advantage in survival compared to patients who underwent only one procedure (3-years 63.2% vs. 38.4%, *p* 0.030; ratio 0.489, 95% CI = 0.24–1.01; Figure 4).

**Figure 5:**
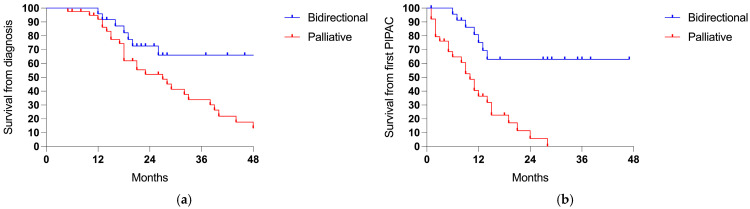
Survival comparing the bidirectional (24 cases) and palliative intent (40 patients): (**a**) the survival from the diagnosis of PSMs (*p* 0.011) and (**b**) the survival from the first PIPAC session (*p* < 0.001).

Patients treated with a bidirectional intent showed longer survival rates from the diagnosis of PSMs (3-years 66.0% vs. 33.9%, *p* 0.011; ratio 0.361, 95% CI = 0.18–0.71; Figure 5a) and from the first PIPAC session (3-years 63.0% vs. 0%, *p* < 0.001; ratio 0.223, 95% CI = 0.11–0.45; Figure 5b).

**Figure 6:**
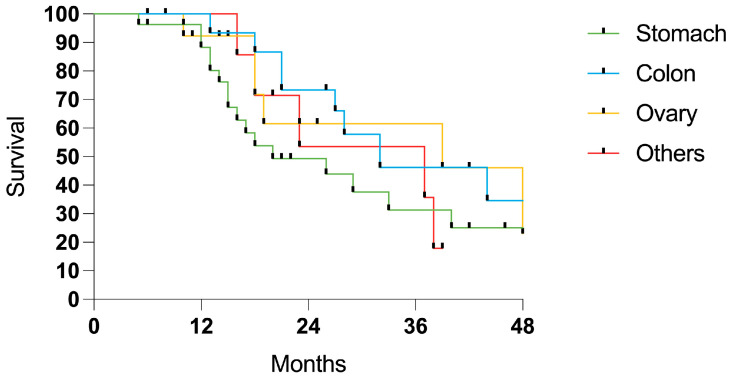
Survival in patients with PSM from gastric (27 cases), colorectal (15 cases), ovarian (14 cases), and miscellaneous sites (8 cases).

When categorized by the tumor origin, colorectal and ovarian cancer showed a trend for a higher survival probability (Figure 6).

Conversion surgery has been pursued in twenty-two (34.4%) cases, including two cases that were initially designated for a palliative treatment approach. This included 10 cases of gastric cancer (conversion rate of 37.0%), 4 colorectal (conversion rate of 26.7%), 6 ovarian (conversion rate of 42.9%), and 2 peritoneal (conversion rate of 66.7%). The mean PCI at the first PIPAC session was 9.5 (IQR 5.25–13.75). Seven patients (31.8%) received two or more sessions of PIPAC in combination with systemic chemotherapy. Within a median follow-up period of 25.5 months, 18 out of 22 converted cases (81.9%) were free of disease recurrence at the last check-up.

### 3.4. PSM from Gastric Cancer

The primary tumor was in the upper third in four patients, the middle third in twelve patients, the lower third in eight patients, and the linitis plastica in three cases. Among the 27 patients, 8 (29.6%) had a Lauren intestinal-type histology, while 19 (70.4%) had diffuse or mixed types. According to the World Health Organization, 68% of cases were classified as the poorly cohesive subtype, and among these, 82.4% contained signet-ring cells.

**Figure 7:**
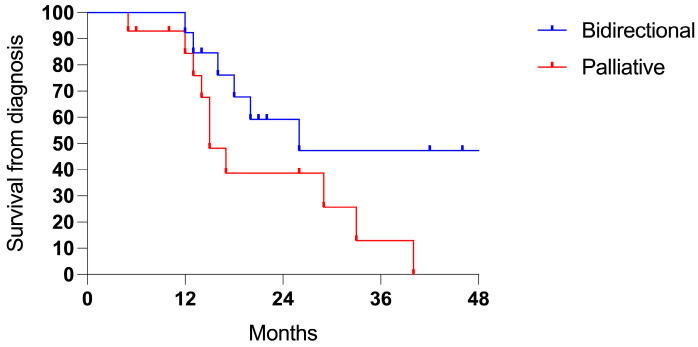
Survival in PMS from gastric cancer (27 cases): bidirectional (13 cases) and palliative intent (14 patients).

Patients who underwent the bidirectional treatment exhibited a trend for improved survival, although this was not statistically significant (3-years 47.4% vs. 21.6%, *p* = N.S.; Figure 7).

## 4. Discussion

The modern management of PSMs involves a multidisciplinary approach integrating systemic and locoregional chemotherapy with the aim of achieving a potentially curative result. Currently, these treatments are limited to a specific patient group [24], and therapeutic strategies are primarily directed towards local disease control and symptom relief [25,26].

PIPAC has emerged as an innovative technique that enhances the bioavailability of chemotherapeutic agents while minimizing systemic toxicity [27]. Early encouraging results have led multiple centers to adopt this option and reserved it for patients deemed unsuitable for CRS and HIPEC as part of preoperative systemic therapy [28,29]. Several clinical trials are currently underway evaluating its efficacy for PSMs arising from gastric cancer, peritoneal mesothelioma, ovarian cancer, and colorectal cancer [30].

The analysis of the data from phase I and II studies indicates that PIPAC is technically feasible in approximately 90% of cases. In the remaining patients, access to the abdominal cavity may be unsuccessful due to adhesions or advanced peritoneal disease [31]. In our experience, only two patients were unable to undergo PIPAC owing to extensive intraperitoneal adhesions, producing a low access failure rate. Furthermore, PIPAC demonstrates a high safety profile, with reported adverse event rates ranging from 0 to 37% [28]. A recent meta-analysis reported CTCAE grade 3 complications in 7% of cases, grade 4 in 0.8%, and grade 5 in 1.6%. The mortality rate ranges from 1% to 8.3% [8,32]. In our series, we observed an overall complication rate of 14.1%, with severe complications accounting for 6.2%. These outcomes likely reflect the careful selection of patients with a sufficient functional reserve and both a physical and emotional resilience to tolerate surgical procedures during ongoing chemotherapy and the exclusion of patients with a rapid progression during chemotherapy.

Existing clinical studies have reported a wide range of clinical response rates: 62–88% in ovarian cancer, 50–91% in gastric cancer, and 71–86% in colorectal cancer [29,33,34]. However, the true survival benefit of this approach is difficult to evaluate due to the heterogeneity of patient populations across studies, differing clinical settings, and the frequent inclusion of patients with advanced diseases who have undergone multiple lines of chemotherapy.

An important consideration is that the median recovery time from the systemic chemotherapy after the PIPAC procedure was approximately 14 days. This indicates the minimal clinical impact on the patients’ functional reserves. This approach, which integrates PIPAC and intravenous chemotherapy, was associated with promising survival trends [35]. In our series, patients treated with the bidirectional approach exhibited significantly improved survival outcomes than those receiving the palliative intent therapy alone. Specifically, the 3-year survival rate from the time of the PSM diagnosis was 66.0% in the bidirectional group and half that in the palliative group. Moreover, conversion surgery was achieved in 22 patients (34.4%), demonstrating a significant downstaging of the disease extension. Two of these patients were initially classified as palliative approaches, which highlights the potential for reclassifying treatment pathways based on favorable responses. Notably, the complete regression of both peritoneal and primary tumors was achieved in two patients. Although it is clear that the two groups had different tumor burdens, which also influenced long-term outcomes, the main aim of this study was to distinguish results, rather than to prove the different efficacies of the bidirectional and palliative approach.

The tumor origin also played a significant role in survival outcomes. Patients with colorectal and ovarian primary tumors demonstrated superior survival when compared to those with gastric cancer. These differences could likely be due to variations in their tumor biology, chemosensitivity, and response to intraperitoneal treatment modalities. However, the limited sample size might be biasing these results.

Approximately half of the patients in our cohort were treated for PSMs of a gastric origin. Of these, 48.1% received a treatment with a bidirectional therapeutic intent. These patients demonstrated a higher survival rate compared to those treated with a palliative intent alone (26 vs. 17 months) and a conversion rate of 37%. This is a very high value compared to the data reported in the literature, especially considering that these patients had peritoneal dissemination (Yoshida 3 or 4 classification) [36].

The phase II PIPAC-GA2 study reported a pathological response in 60% of patients treated with PIPAC using cisplatin and doxorubicin in combination with systemic XELOX chemotherapy. Despite this promising response rate, the mean overall survival was 13 months, and no cases of conversion to curative surgery were observed [37]. Conversely, a recent systematic review encompassing 53 studies and a total of 4719 PIPAC procedures performed in 1990 patients highlighted key findings related to the bidirectional treatment approach. The analysis, which focused on European studies published between 2018 and 2023, reported conversion rates ranging from 0% to 26% among patients treated with combined intraperitoneal and systemic chemotherapy. Notably, the review also identified a positive association between the number of PIPAC sessions and survival. Specifically, the median survival increased from 4.7 to 10.5 months in patients receiving a single PIPAC session to 15–16 months in those who underwent two or more sessions, suggesting a potential cumulative benefit of repeated intraperitoneal administrations [34]. Similarly, we obtained a median survival of 23.5 months compared to 18 months in patients undergoing only one PIPAC procedure.

The role of PIPAC in gastric cancer has been extensively explored in specific clinical scenarios. These include patients with disease progression following multiple lines of systemic chemotherapy [38], those presenting with extraperitoneal metastases [39], and individuals with a high PCI [40]. Conversely, data on the prophylactic approach in patients at high risk of developing metachronous peritoneal metastases are still limited. The PIPAC-OPC4 study investigated the use of PIPAC in patients with advanced gastric cancer, a poorly cohesive (signet-ring cell) histology, or a positive peritoneal cytology who received PIPAC immediately following a curative-intent surgery. The study reported a surgical reintervention rate (ClavienDindo grade 3b) of 23.8% [41].

Limitations of the present study include the retrospective design, the relatively low sample size, and, above all, the lack of a control group. Furthermore, a potential bias due to the patient selection cannot be excluded. Regardless, this study could provide new insights towards the adoption of the bidirectional strategy and the inclusion of PIPAC in the multimodal management of PSMs.

**Figure 8:**
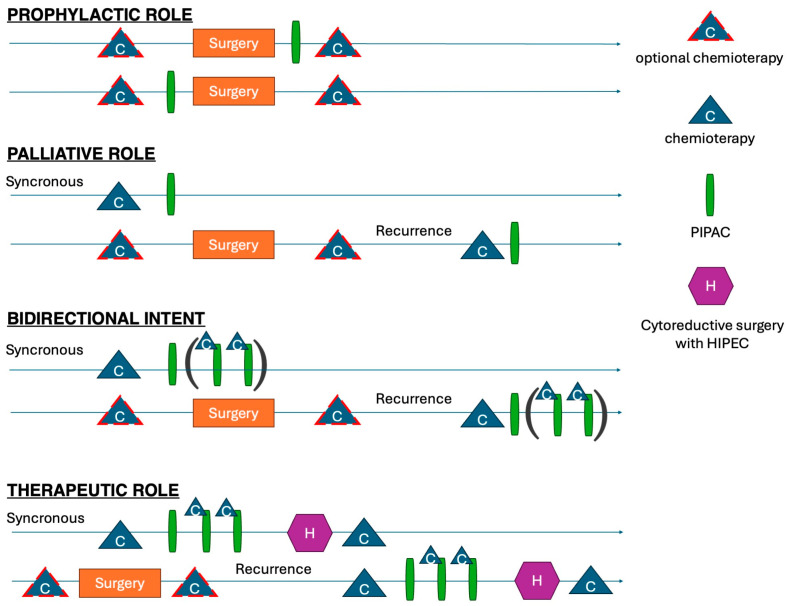
Different potential roles of PIPAC in multimodal approach to PSM.

Prospective studies focusing on the therapeutic or prophylactic roles are eagerly awaited to define the precise indications, optimal timing, and future perspectives (Figure 8) [42]. In line with Sugarbaker’s argumentation [43], complex procedures should be regarded as experimental treatments, performed by high-volume surgeons in high-volume institutions to minimize postoperative morbidity and mortality. The phase-III PIPAC VER-ONE randomized control trial will aim to compare systemic chemotherapy alone vs. a combined intraperitoneal and intravenous therapy in oligometastatic patients with a PCI equal or less than six and/or a positive peritoneal cytology [44]. That study is ongoing.

## 5. Conclusions

PIPAC is a safe and feasible treatment when performed in specialized centers. It may be considered an additional therapeutic option for patients with extensive peritoneal disease who are not eligible for CRS or HIPEC. The minimal clinical impact on patients provides a compelling argument for its integration into a bidirectional strategy, potentially serving as a bridge to conversion surgery. However, careful patient selection is essential to avoid treating individuals with a poor performance status or rapidly progressive disease.

## Figures and Tables

**Table 1:** Clinical characteristics in the bidirectional and palliative groups.

(%)	Entire Series (64)	Bidirectional (24)	Palliative (40)	*p*
Location				-
Gastric	27 (42.2)	13 (48.1)	14 (51.9)	
Colorectal	15 (23.4)	3 (20.0)	12 (80.0)	
Ovarian	14 (21.9)	6 (42.9)	8 (57.1)	
Pancreatic	4 (6.2)	0	4	
Breast	1 (1.6)	0	1	
Mesothelioma	3 (4.7)	2 (66.7)	1 (33.3)	
PCI at surgery, IQR	14 (9–25)	9.5 (5–14)	23 (14–35)	<0.001
Ascites	39 (60.9)	10 (25.6)	29 (74.4)	0.030
Cytology+	31 (48.4)	11 (35.5)	20 (64.5)	0.889
PIPAC Sessions				0.233
1	48 (75)	16 (33.3)	32 (66.7)	
2 or more	16 (25)	8 (50)	8 (50)	
Complications	9 (14.1)	3 (33.3)	6 (66.7)	0.839
CTCAE grade I	5 (7.8)	2 (40)	3 (60)	
CTCAE grade II	1 (1.6)	0 (0)	1 (100)	
CTCAE grade III	3 (4.7)	1 (33.3)	2 (66.7)	
30-day mortality	2 (3.1)	0 (0)	2 (100)	0.734

**Table 2:** Median survival months (3-year survival rate) stratified by tumor origin.

	All Series Median (3-y)	Bidirectional Median (3-y)	Palliative Median (3-y)	*p*
Entire series	32 (44.6%)	24 (66.0%)	19 (33.9%)	0.011
Stomach	20 (31.3%)	26 (47.4%)	17 (21.6%)	0.188
Colon	32 (46.2%)	27 (0)	30 (40.0%)	0.560
Ovary	39 (61.5%)	24 (80.0%)	18 (42.9%)	0.076
Others	37 (53.6%)	38 (100%)	26.5 (53.3%)	0.480

## Data Availability

The data presented in this study are available on request from the corresponding author due to privacy concerns.

## Abbreviations

The following abbreviations are used in this manuscript:

PSM	Peritoneal Surface Malignancies
PIPAC	Pressurized Intraperitoneal Aerosol Chemotherapy
CRS	Cytoreductive Surgery
HIPEC	Hyperthermic Intraperitoneal Chemotherapy
ECOG	Eastern Cooperative Oncology Group
PSS	Prior Surgical Score
PCI	Peritoneal Cancer Index
RECIST	Response Evaluation Criteria in Solid Tumors
CTCAE	Common Terminology Criteria for Adverse Events
OS	Overall Survival
SD	Standard Deviation
IQR	Interquartile Range
